# The Association of Persistent Symptoms of Depression and Anxiety with Recurrent Acute Coronary Syndrome Events: A Prospective Observational Study

**DOI:** 10.3390/healthcare10020383

**Published:** 2022-02-17

**Authors:** Abdullah S. Alhurani, Ayman M. Hamdan-Mansour, Muayyad M. Ahmad, Gabrielle McKee, Sharon O’Donnell, Frances O’Brien, Mary Mooney, Zyad T. Saleh, Debra K. Moser

**Affiliations:** 1 School of Nursing, The University of Jordan, Amman 11942, Jordan; a.alhurani@ju.edu.jo (A.S.A.); mma4@ju.edu.jo (M.M.A.); zyad.saleh@ju.edu.jo (Z.T.S.); 2 Department of Physiology, School of Nursing and Midwifery, D02 PN40 Dublin, Ireland; gmckee@tcd.ie (G.M.); sodonne@tcd.ie (S.O.); obrienfr@tcd.ie (F.O.); mooneyma@tcd.ie (M.M.); 3 College of Nursing, University of Kentucky, Lexington, KY 40503, USA; dmoser@uky.edu

**Keywords:** acute coronary syndrome, depression, anxiety

## Abstract

The purpose of this study was to examine the role of persistent symptoms of depression and anxiety in a second acute coronary syndrome (ACS) event. Data presented in this study were from an RCT study. A follow-up for 24 months after baseline to detect a second ACS event among 1162 patients from five hospitals. Hierarchal Cox regression analyses were used. The results showed that persistent depression only (HR 2.27; 95% CI: 1.35–3.81; *p* = 0.002), and comorbid persistent depression and anxiety (HR 2.03; 95% CI: 1.03–3.98; *p* = 0.040) were the significant predictors of a second ACS event. Secondary education level compared to primary educational level (HR 0.63; 95% CI: 0.43–0.93; *p* = 0.020) and college or more education level compared to primary educational level (HR 0.47; 95% CI: 0.27–0.84; *p* = 0.011) were the only demographic variables that were significant predictors of a second event. The study reveals that attention must be paid by healthcare providers to assess and manage persistent depression; particularly when it is co-morbid with anxiety.

## 1. Introduction

Coronary artery disease is the number one killer in the world [1]. Acute coronary syndrome (ACS) is an umbrella term used to cover any condition that reduces blood flow to cardiac muscle and leads to signs and symptoms of acute myocardial ischemia [1]. ACS includes unstable angina, non-ST elevation myocardial infarction (NSETMI), and ST-elevation myocardial infarction (STEMI) and these conditions may progress to serious complications like heart failure or death [2]. Caring of individuals with ACS requires understanding of undermining reasons and antecedents that may include psychological ones. Recent studies also have emphasized the role of gender, physical fitness, obesity, age, and therapeutic regimens in developing ACS [3,4]. Such factors also vary depending on other intrinsic and biological factors that are associated with and influenced by medication and treatment protocol used following acute cardiac injury [5]. Therefore, the connection between psychological factors such as depression and anxiety might act reciprocally with ACS either causing or jeopardizing the occurrence and consequences of second ACS [6].

Depression is a serious mental illness that interferes with individual’s ability to function normally, socially and occupationally. Studies showed that depression is becoming prevalent among patients with coronary artery diseases. It has been reported that 20% of patients admitted to hospital with ACS meet the criteria of major depression symptoms reported by DSM-5 [7]. Such percentage might be increased in patients with ACS [7]. Although studies linked depressive symptoms to ACS morbidity and mortality rates [7,8,9], the co-occurrence of depression and anxiety is not well addressed [1]. Connecting the co-occurrence of depression and anxiety among patients with ACS is assumed to compromise patients’ wellbeing and recovery due to assumed social and occupational dysfunctions that might also occur due to anxiety [10,11,12]. However, addressing the persistent state of depression and anxiety among patients with ACS is rarely discussed, as well [10,11,12]. In such situations, the co-occurrence of depression and anxiety in patients with ACS provides more complex understanding for a second ACS. The notion that physical symptoms create psychological response, and that psychological responses would exacerbate the physical symptoms might be applicable in such situations [13,14]. Readmission and depression have been reported for those with ACS in the ensuing two years [13]. Such connection has been also reported with number of limitations including adherence, dieting, and psychological counseling [13]. On the other hand, other studies have indicated that anxiety and depression have been reported prior the first ACS [15]. The question remains whether anxiety and depression did contribute to first and second ACS or the consequences of physical symptoms of the first ACS did cause the occurrence of a second ACS [16]. However, there is limited information in the literature regarding the association of persistent depression and anxiety with recurrent ACS. This study provides an understanding for such an issue. Thus, the purpose of this study was to investigate the role of persistent symptoms of depression and anxiety in second ACS event. The specific aims were:

- To assess the persistent anxiety and depression among patients with second ACS event.
- - To identify the relationship between persistent anxiety and depression and second ACS event.

## 2. Materials and Methods

Data presented in this study were from a randomized controlled trial called ACS Delay Time Intervention Trial [17]. The trial examined the effect of an educational intervention on knowledge attitudes, beliefs, and the pre-hospital delay time in patients diagnosed with ACS [12,17]. Patients’ enrollments took place in five hospitals (N = 1162). Patients were recruited from coronary care units, and cardiology floors after eligibility screening had been done by nurses who worked in these hospitals. Patients have been clustered to describe the presence of symptoms of depression and anxiety at baseline. A cut-off point has also been created at 75th percentile. This process was repeated at three months follow-up period. Patients were clustered, then, into four groups according to existence of persistent depression and anxiety as follows: (1) none, (2), had persistent depression only; (3) had persistent anxiety only, and (4) has both persistent depression and persistent anxiety. Those who have been diagnosed with ACS, undergoing discharge were considered eligible for participation (See flow diagram 1). While those institutionalized, diagnosed with serious cognitive or physical disability that interferes with their ability to comprehend, understand, and answer the question, and those with serious medical complications were excluded from the study. Prior to being included in the study, information about the study and its significance were discussed with those who expressed interest in participation. The information was introduced in verbal and written format. Eligible and interested candidates were recruited by a research nurse who obtained signed consent and collected data through interviews and medical record review. Once informed consent signed and the patients are clinically stabilized according to primary caregivers, data were collected prior discharge and then follow-up over the phone. Those who have been readmitted to the unit were followed up and checked to confirm the second ACS.

Symptoms of depression and anxiety were measured using The Multiple Adjective Affect Checklist (MAACL) 15. The MAACL contains 132 alphabetically organized adjectives. Patients were asked to select those adjectives that described how they felt. Those with higher scores are more likely to suffer anxiety and depression. The 75^th^ percentile has been created as a cutoff point for those classified with anxiety or depression. The scale has good internal consistency, stability, construct, and concurrent validity. Cronbach’s alpha for the MAACL was reported to be 0.82 for the Anxiety Scale and 0.81 for the Depression Scale [18,19].

To measure for the second ACS, a month-based follow-up phone call and electronic and non-electronic chart reviews were used to determine dates for hospitalizations. Patients were followed for at least 24 months after baseline to detect second ACS events. Demographic and clinical information were also collected at baseline from patients along with their medical records by interviewing patients and by reviewing their electronic and non-electronic charts. These variables include age, gender, marital status, education level, smoking history, body mass index (BMI), hypertension history (HTN), and diabetes history (DM).

The patients were informed that participation is strictly voluntary and deciding not to take part in the study would not pose any disadvantage to them. The participants were informed that the information they share in the survey are confidential and the data would be locked in a password protected folder in a PC of the investigators. The work described was carried out in accordance with The Code of Ethics of the World Medical Association (Declaration of Helsinki) for experiments involving humans; Uniform Requirements for manuscripts submitted to biomedical journals.

Data were analyzed using the IBM-SPSS 25. Descriptive statistics (Mean, median, model), dispersion measures (SD, variance), and interpercentile measures were used to describe the variables of the study. *t*-test, ANOVA, and chi-square were used to compare the group differences.

Hierarchal Cox proportional regression analyses were used to determine whether co-morbid persistent symptoms of depression and anxiety, independently, predicted the second ACS event. Data were entered into the regression in three blocks. The following covariates were considered: age, gender, marital status, education level, smoking history, BMI, HTN, and DM. In the first block, demographic variables (i.e., age, gender, marital status and education level) were entered. In the second block, clinical variables (i.e., smoking history, BMI, HTN, and DM)) were entered. In the final block, depression and anxiety group was entered into the model. The assumptions of hierarchal Cox proportional hazards modeling were tested, and no violations occurred. *p*-value was set at 0.05.

## 3. Results

### 3.1. Demographic Characteristics

Demographic and clinical characteristics of patients (N = 1162) are summarized in Table 1. The mean level of depression and anxiety at baseline were 10.29 ± 3.69, 18.70 ± 4.46, respectively. The mean level of depression and anxiety decreased at three months follow-up period to 7.97 ± 4.40 and 16.13 ± 6.29, respectively. A total of 74 (7.0%) patients had persistent depressive symptoms only, 47 (4.4%) had persistent anxiety symptoms only, 56 (5.3%) had co-morbid persistent symptoms of depression and anxiety, and the remaining 880 (83.3%) had no present anxiety or depression. A total of 136 (11.3 %) participants had a second ACS event described as the following: 6 participants (0.5%) had STEMI, 14 participants (1.2%) had NSTEMI, and 116 participants (9.7%) had unstable angina.

### 3.2. Regression Analysis

Hierarchal Cox regression was run for the depression and anxiety group treated as a categorical variable and second ACS event as the outcome. In the first block, demographic variables (i.e., age, gender, marital status, and education level) were entered. In this block, only secondary education level compared to primary educational level (HR 0.60; 95% CI: 0.40–0.88; *p* = 0.009) and college or higher compared to primary educational level (HR 0.44; 95% CI: 0.25–0.78; *p* = 0.005) were significant predictors of second ACS event.

**Figure 1:**
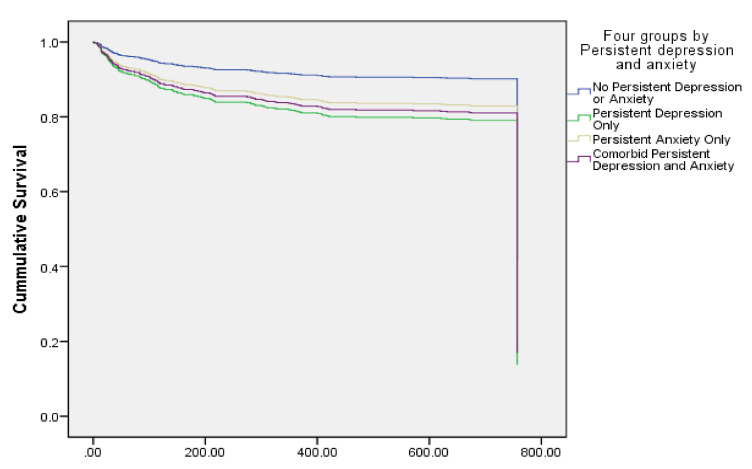
Acute coronary syndrome second event according to comorbid depression and anxiety.

In the second block, clinical variables (i.e., smoking history, BMI, HTN, and DM) were entered. Secondary education level compared to primary educational level (HR 0.60; 95% CI: 0.41–0.89; *p* = 0.011) and college or more education level compared to primary educational level (HR 0.45; 95% CI: 0.26–0.81; *p* = 0.007) remained the only significant predictors of second ACS event. In the final block, depression and anxiety group was entered into the model. The final model demonstrated that secondary education level compared to primary educational level (HR 0.63; 95% CI: 0.43–0.93; *p* = 0.020), college or more education level compared to primary educational level (HR 0.47; 95% CI: 0.27–0.84; *p* = 0.011), persistent depression only (HR 2.27; 95% CI: 1.35–3.81; *p* = 0.002), and comorbid persistent depression and anxiety (HR 2.03; 95% CI: 1.03–3.98; *p* = 0.040) were the significant predictors of second ACS event. Patients with persistent depression are 2.27 times more likely to have a second ACS event compared to those who do not have persistent depression or anxiety. In addition, patients with comorbid persistent depression and anxiety are 2.03 times more times more likely to have a second ACS event compared to those who do not have persistent depression or anxiety (Table 2, Figure 1).

**Figure 2:**
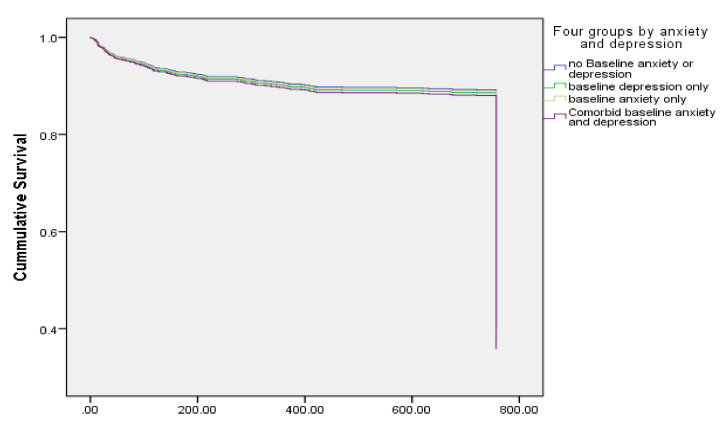
Acute coronary syndrome second event according to comorbid depression and anxiety.

A second analysis was conducted using the hierarchal Cox regression with baseline depression and anxiety. Variables were entered in three blocks just as the previous analysis. In all three blocks, secondary education level compared to primary educational level (HR 0.59; 95% CI: 0.40–0.857; *p* = 006) and college or more education level compared to primary educational level (HR 0.50; 95% CI: 0.29–0.86; *p* = 0.013) were the only significant predictors of ACS second event. None of the four depression and anxiety variables was a significant predictor of ACS second event in this analysis and the results were as following: depression only (HR 1.06; 95% CI: 0.64–1.75; *p* =0.814), anxiety only (HR 1.03; 95% CI: 0.58–1.83; *p* = 0.926), comorbid depression and anxiety (HR 1.12; 95% CI: 0.70–1.78; *p* = 0.646) (Figure 2). Thus, persistent depression and anxiety but not depression and anxiety at baseline are associated with the second ACS event.

## 4. Discussion

The comorbidity of psychological and medical problems is no more a discussion point, and rather, a point of agreement. Researchers are almost in agreement with that a reciprocal relationship exists between medical and psychological problems [20,21]. We found in this study that persistent depression and comorbid persistent depression and anxiety were statistically significant predictors of a second ACS event; persistent depression was the strongest predictor. Theoretically, it would appear that comorbid persistent depression and anxiety would have a stronger effect than persistent depression only. However, the combination of comorbid persistent depression and anxiety was the second strongest predictor for the second ACS. Anxiety alone was not a significant predictor, which could explain that anxiety did not add power to the prediction of second ACS; on the contrary, it has attenuated the effect of depression. The results do support previous reports that persistent symptoms depression and anxiety has increased the risk of mortality in patients with cardiac problems [15,22]. One explanation is that depression and anxiety symptoms such as insomnia, loss of energy, weight issues, decreased pain tolerance, headache, and gastrointestinal problems might have added to cardiogenic factors related to ACS. The significant role of depression in second ACS, however, indicates the overlap and exacerbates the effect of psychosomatic problems on the patients’ wellbeing. In other words, the origin of symptoms whether due to depression or to cardiac effect might add to patients’ burden of disease causing further psychosomatic effect that contributed to second ACS. Such an explanation supports the notion that a second ACS event is likely a physiological sequels of the maladaptive behaviors associated with depression [23]. Worth to add here that factors that might also contribute to second ACS might be patient-related. For example, depressed patients are more reluctant to comply with suggested care protocols, attend follow-up visits, or make behavioral changes that could insulate them from second ACS events [7]. In addition, those patients are more likely to withdraw prescribed medications that affects efficacy of drugs and the intended outcomes of treatment protocol. It has been found that young and old people and being female are associated with lack of adherence among patients post cardiac infarction [24]. This might also confirm the significant contribution of depression while anxiety was not an influencing factor.

The literature emphasized the role of depression in developing serious complications and mortality in patients with ACS [7]. The risk of ACS recurrence or even mortality is high among patients whose depressive symptoms are persist or resistant to treatment [7,14,15]. Therefore, management of cardiac problems requires that healthcare professionals work collaboratively to address psychological and physiological underlying reasons related to ACS upon the first admission and making a critical follow-up to address the influence of depressive symptom on receiving appropriate care to prevent second ACS. Furthermore, with regard to the high risk of second ACS reported in this study, patients with persistent depression are about 2.3 times more likely to have a second ACS attack compared to those who do not have, do add to the body of knowledge counteracting the treatment guideline proposed by the American Heart Association which does not include depression as risk factor for poor prognosis among patients with cardiogenic problem [7,14]. Our results do support the very recent study that risk of heart disease are much higher (up to 2.5 fold) among patients with psychiatric disorders compared to those who are not. Although persistent depression and anxiety does not count as psychiatric disorders, the severe forms might increase the vulnerability to psychiatric illness and further psychosomatic problems [25]. One significant issue to raise here related to variation in report that connects psychological problems and cardiac disease is related to approaches used to identify psychological and psychiatric disorders. Psychiatric disorders need to be confirmed through clinical observation of criteria advised by DSM-5 for example. However, patients may have some of these criteria and still not diagnosed which might be described as depressive symptomatology. Such an issue is ignored in the literature where depression has been identified as a disorder or just a group of depressive symptoms. Nevertheless, untreated depressive symptoms might progress to disorders causing more harm to patient. This indicates that health care professionals including nurses need to be aware of the negative effect of depressive symptoms and its severity which might be non-diagnosable but might exacerbate to influence the patient’s wellbeing causing second ACS.

Regarding the sociodemographic factors, we found that education was a significant predictor, while gender and age were not. This indicates that the role of sociodemographic factors in second CS is minimal compared to psychological factors. Although age and gender play a significant role in defining severity of chest pain, their role as predictors of second ACS is not significant supporting previous findings in this field [7,23,26].

One limitation of this study is related to data collection process in which more unstructured format of data collection regarding depression and its role in adherence to medical regimen among patient with cardiac problems might reveal more information and better understanding. Another limitation is related to the lack of comparative data with patients without ACS that might reveal more informative results.

## 5. Conclusions

This study attempted to investigate the role of persistent depression and anxiety in second ACS. We found that second ACS is common and prevalent among those with depression and less with those of anxiety. Moreover, educated people showed more positive outcomes, and age and gender are not associated with second ACS. The study infers a need to manage depression among patients with ACS to prevent second ACS due to the significant role of depression in lack of adherence to medical regiment after discharge. The study has implications to nurses in cardiology units and to all other psychology and healthcare professionals. Cardiology nurses need to connect psychological antecedents of cardiac problems and the higher risk for second ACS among those with depression and anxiety. Thus, assessment of psychosomatic signs and symptoms has to be a priority and should be integrated in the routine nursing care plans for patients admitted with ACS. Follow-up care also has to emphasize the signs and symptoms of depression and anxiety as one proposed measure to prevent second ACS and due to the significant role of depression in lack of adherences and second ASC. Managing psychological problems related to cardiac problems is also a priority. Future investigators should attempt to determine why persistent depression alone is a stronger predictor of a second ACS event.

## Figures and Tables

**Table 1:** Sample baseline characteristics (N = 1162).

Characteristic	N (%) or MEAN ± SD
Age	63.45 ± 11.90
Anxiety score (baseline)	10.29 ± 3.69
Anxiety score (3 months)	7.97 ± 4.40
Depressive symptoms score (baseline)	18.70 ± 4.46
Depressive symptoms score (3 months)	16.13 ± 6.29
Anxiety and depression categories	
No persistent Anxiety or Depression	880 (83.3)
Persistent depression Only	74 (7.0)
Persistent anxiety Only	47 (4.4)
Co-morbid persistent anxiety and depression	56 (5.3)
	
BMI	
	846 (72.8)
	316 (27.2)
Marital status	
Single/separated/widowed/divorced	396 (34.1)
Married/ living with someone	766 (65.9)
Education Level	
Little or primary level completed	417 (35.9)
Secondary level completed	523 (45.0)
College or higher	222 (19.1)
Current smoker	575 (49.5)
HTN	682 (58.7)
Diabetes mellitus	190 (16.3)

**Table 2:** Cox proportional hazard regression of variables associated with ACS second event, and anxiety and depression treated as categorical variables.

Predictor Variables	Odds Ratio	95% CI	P
Block I			
Age	1.00	0.98–1.01	0.617
Female Gender	1.41	0.97–2.06	0.075
Secondary education compared to primary	0.60	0.40–0.88	0.009
College or more education compared to primary	0.44	0.25–0.78	0.005
Married/living with someone compared to single/separated/widowed/divorced	0.79	0.55–1.15	0.220
Block II			
Age	1.00	0.98–1.01	0.725
Female Gender	0.92	0.95–2.03	0.092
Secondary education compared to primary	0.60	0.41–0.89	0.011
College or more education compared to primary	0.45	0.26–0.81	0.007
Married/living with someone compared to single/separated/widowed/divorced	0.79	0.54–1.15	0.220
Smoker compared to non-smoker	1.00	0.69–1.44	0.977
Hypertensive compared to not hypertensive	1.20	0.81–1.75	0.362
Diabetic compared to not diabetic	0.92	0.57–1.50	0.743
BMI	1.02	0.98–1.05	0.438
Block III (Final Model)			
Age	1.00	0.98–1.02	0.944
Female Gender	0.31	0.83–1.81	0.311
Secondary education compared to primary	0.63	0.43–0.93	0.020
College or more education compared to primary	0.47	0.27–0.84	0.011
Married/living with someone compared to single/separated/widowed/divorced	0.81	0.56–1.18	0.278
Smoker compared to non-smoker	0.96	0.66–1.39	0.821
Hypertensive compared to not hypertensive	1.19	0.81–1.75	0.388
Diabetic compared to not diabetic	0.87	0.53–1.41	0.560
BMI	0.47	0.98–1.05	0.470
Persistent depression Only	2.27	1.35–3.81	0.002
Persistent anxiety Only	1.81	0.89–3.66	0.100
Comorbid Persistent anxiety and depression	2.03	1.03–3.98	0.040
Overall Model (χ^2^ = 37.14, df. = 12; *p* < 0.001)			
